# Integrative characterization of host–microbiome-diet axes during early-life development of the murine gut

**DOI:** 10.1186/s40168-026-02541-3

**Published:** 2026-10-06

**Authors:** Giacomo Carta, Feng Xian, Daniel Malzl, Manuela Schmidt, David Gómez-Varela

**Affiliations:** 1 Division of Pharmacology and Toxicology, Department of Pharmaceutical Sciences, Faculty of Life Sciences, University of Vienna, Vienna, Austria; 2 Center of Excellence for Metaproteomics, University of Vienna–Bruker Daltonics, University of Vienna, Vienna, Austria; 3 CeMM Research Center for Molecular Medicine of the Austrian Academy of Sciences, Vienna, Austria; 4 Ludwig Boltzmann Institute for Network Medicine at the University of Vienna, Vienna, Austria

**Keywords:** Gut microbiome, Mouse development, Host-microbiome interactions, Diet, Metaproteomics

## Abstract

**Background:**

Early-life development of the gut microbiome plays a critical role in shaping host physiology. However, a comprehensive understanding of how diet, microbial community assembly, functional capacity, and host intestinal maturation axes evolve and coordinate over time remains lacking. Most studies focus on a single axis, rely on cross-sectional sampling, or have limited functional resolution, restricting insight into developmental dynamics. Here, we characterized early-life maturation of the gut ecosystem using longitudinal, metaproteome-level analysis in a murine model.

**Results:**

Using metaproteomics, we profiled fecal samples collected at seven postnatal time points from day 10 to weaning and into early adulthood (day 48) in pups from two contemporaneously raised C57BL/6 cohorts differing only in maternal origin (a long-established local colony and newly purchased pregnant females from the same vendor). Analyses accounted for time, cohort, and sex effects. Microbial communities underwent pronounced taxonomic succession, shifting from early dominance by facultative anaerobes to obligate anaerobes after weaning, accompanied by increasing species richness and functional complexity across cohorts and sexes. Taxonomic changes were co-occurring with an increase in functional redundancy that converged by postnatal day 34 and remained stable into early adulthood. KEGG clustering revealed patterns consistent with changes in metabolic pathway representation alongside maintenance of core functions.

Direct detection of low-abundant dietary proteins provided evidence for dietary transitions coinciding with microbial maturation: milk proteins were detected only before weaning, while solid food components predominated over time. Maternal origin was significantly associated with microbial engraftment trajectories, leading to cohort-specific taxonomic and functional differences despite identical housing and diet. In parallel, host intestinal proteome maturation mirrored microbial succession, with concomitant shifts in metabolic, absorptive, regulatory, and effector pathways, including antimicrobial peptides and carbohydrate-modifying enzymes.

**Conclusions:**

By directly integrating microbial, dietary, functional, and host axes within a longitudinal framework, this study provides a comprehensive view of murine gut ecosystem maturation during early life and offers a reference for interpreting developmental microbiome dynamics and improving experimental design and reproducibility in mouse studies.

Video Abstract

**Supplementary Information:**

The online version contains supplementary material available at https://doi.org/10.1186/s40168-026-02541-3.

## Background

The gut microbiome plays a central role in mammalian development, shaping host physiology through effects on immune maturation, nutrient metabolism, and organ development [1–3]. Early life represents a critical window during which gut microbial communities are established and refined [4–7], with long-term consequences for health and disease susceptibility [7–13]. Despite extensive interest in early-life microbiome development, how microbial community assembly, functional maturation, and host intestinal development are coordinated over time is unknown.

Most current knowledge of gut microbiome development derives from genomic and amplicon-based studies [12, 14–17], which primarily describe taxonomic composition and, consequently, infer functional potential. While these approaches have been instrumental in defining broad patterns of microbial community assembly dynamics, they do not directly capture expressed microbial functions or host physiological responses [11, 18]. Moreover, many studies rely on cross-sectional sampling or limited temporal resolution [15, 19, 20], restricting insight into dynamic host–microbiome-diet interactions during key developmental transitions such as weaning. As a result, the functional mechanisms underlying diet, early microbial colonization, host intestinal maturation, and ecosystem-level functional stabilization are only poorly characterized.

Murine models, particularly C57BL/6 mice, are widely used to study microbiome-associated processes relevant to human health, owing to their genetic tractability and substantial overlap in microbial functional capacity with humans [21]. Beyond host genetics and diet, early-life microbial assembly is also shaped by vertical transmission from the mother [10, 15, 16]. Maternal microbiota have emerged as key biological determinants of early microbial engraftment, influencing both the initial composition and the subsequent developmental trajectory of the gut ecosystem [10, 11].

At the same time, accumulating evidence indicates that gut microbiota composition in C57BL/6 mice varies across vendors, breeding colonies, and housing conditions, introducing a significant source of experimental variability [21–24]. Differences in maternal microbiota associated with animal sourcing and colony history can therefore imprint cohort-specific microbial trajectories in offspring, even under otherwise standardized experimental conditions. Yet, the extent to which such maternal-origin effects are reflected over early development, and whether they translate into functional signatures in the adult pup’s gut ecosystem, is unknown. This uncertainty has important implications for the implementation, reproducibility, and interpretation of mouse-based studies.

Dietary transitions represent another major driver of microbiome maturation during early life [3, 25, 26]. In both humans and mice, the shift from maternal milk to solid food coincides with marked changes in microbial composition and metabolic activity [27, 28]. However, the timing and extent of early exposure to solid food components, and how these dietary inputs intersect with microbial succession and host intestinal responses, have been difficult to assess directly due to sensitivity limits.

To address these gaps, we applied metaproteomics [29] to profile gut microbiome and host intestinal development in mice across seven postnatal time points, from early colonization through weaning and into early adulthood. Using non-invasive and sterile fecal sampling, we followed pups from two contemporaneously raised C57BL/6 cohorts that differed only in maternal origin—a long-established local colony and newly purchased pregnant females from the same vendor—while systematically accounting for sex-specific effects. This integrated approach enabled the detection of very low-abundant dietary proteins, species-resolved microbial profiling, direct measurement of expressed microbial functions, and parallel assessment of host intestinal proteome maturation. By capturing microbial, dietary, functional, and host axes within a single longitudinal framework, this study provides a comprehensive view of how developmental programming of the murine gut ecosystem emerges during early life.

## Methods

### Mice

All the experiments included in this publication were approved by the University of Vienna’s Institutional Animal Care and Use Committee (IACUC; protocol n. 2023-016; February–June 2023) and carried out in compliance with the rules provided in the Guide for the Care and Use of Laboratory Animals, and EU-Directive for the protection of animals used for scientific purposes. Additionally, this work was reported according to the ARRIVE guidelines.

To explore changes in gut microbiota–host development, eight pregnant C57BL/6 J females (8–9 weeks old) were enrolled in the study: four locally bred (L) mice and four purchased from Charles River (CR). The mice labeled as “Local” mothers and pups were obtained from the breeding colony established at the University of Vienna in October 2021 using founder parents obtained from the same vendor from which the new mothers (CR) were purchased. To minimize the impact of environmental factors on the evolution of the microbiota-host development, the mothers and pups from CR and our L colonies were housed in the same room on two different racks under the same humidity and temperature [23]. All mice had ad libitum access to chow (SM VRF1—breeding diet fortified, autoclavable/γ-irradiated, ssniff Spezialdiäten GmbH) and water, under 12:12 light/dark cycle, with light starting at 7:00 AM. Each transparent polycarbonate cage was provided with corncob bedding, and a standardized enriched environment consisting of a red transparent plastic nest box, two wooden tunnels (15 × 5 × 5 cm), and a handful of crinkled natural paper strands for building a high-quality nest [30]. Every Monday, the mice were moved into a cleaned cage with the autoclaved enrichment described above. The cage was changed the following week only if the mother delivered pups on Monday to avoid stress-related behaviors of the mother. Peers of the same sex, from the same cage, were co-housed in the same cage at a maximum of 5 mice per cage. All mice were weaned at 21 days after birth. No mice were single-caged after weaning. No study-specific humane endpoints were established because none of the procedures adopted were invasive, painful, or expected to cause distress to the animals. However, all animals underwent daily health and welfare assessments by trained animal facility personnel. In the event of signs of pain, distress, or compromised welfare, appropriate measures were taken in accordance with the facility’s standard operating procedures, which are based on Directive 2010/63/EU (Article 13.3) and the NC3Rs Humane Endpoints Guidelines. Based on the significant detectable difference in Bray–Curtis index between adult male and female C57BL/6 J mice obtained from different vendors, as shown by Long and coworkers [22, 31], we used a total sample size of 32 animals (7 for sex/group + 1 possible dropout) with an effect size of *η*^2^ = 0.14 (Power [1-β] = 0.95 and *α* = 0.05) with an actual power of 0.969. Bray–Curtis β-diversity at the Species level was considered as the primary outcome measure for the purpose of sample size estimation. The other outcomes listed below were considered secondary and exploratory endpoints.

### Assay strategies and exclusion criteria

The same male experimenter performed the fecal samples (FS) collection from pups and mothers from the L and the CR cohorts [32–34]. The tools and the experimenter’s hands were disinfected with water and 70% ethanol between each animal manipulation. The experimental groups were assessed randomly (Microsoft Excel random list) at each time point from 8 to 12 AM. From Day 8 (D8), all the pups were tail-marked with a surgical pen, and the marks were renewed every 2 days [35, 36]. Pups were a priori excluded from the experiment if they did not provide FS after 2 collection attempts per day for 2 consecutive days. For this reason, to reach the above-mentioned sample size of 32 animals, we used a total of 42 females (18 CR and 28 Local) and 35 males (13 CR and 22 Local) were used to assure reliable fecal sample amount.

### Fecal samples collected from pups and mothers

The FSs from pups were collected as soon as possible after birth. FSs were collected with an interval of at least 2 days between one collection and the other, since a smaller time interval provided no collection of fecal pellets. The FS obtained from the mothers (4 from each group) was collected 26 days after the pups’ delivery (5 days after weaning to minimize the mother stress consequences on pups during lactation) [37, 38]. This way, microbial community similarity structure and baseline values for the offsprings’ microbial composition were provided [15].

The FSs were collected by stimulating the anogenital reflex, mimicking the maternal licking to stimulate defecation and urination in the mice [39, 40]. The pups were stimulated with soft repeated light strokes (cranio-caudal direction) on the perianal region with a wet cotton swab. This technique was adopted for all the animals at each time point. During the stimulation, a sterile plastic 1.5-ml tube was positioned below the mouse tail, and the FSs were collected from the animal by gravity to reduce as much as possible urine and fur contamination.

Fresh FSs were collected in the morning (8–12 AM), placed immediately in autoclaved 1.5-ml plastic sterile tubes pre-cooled on ice, and stored at −80 °C.

The results reported in this study were obtained from a total of 16 females (8 CR and 8 L) and 16 males (8 CR and 8 L). Although the experimental design initially included 224 planned samples from the pups tested, some animals did not produce a fecal pellet at specific time points. Consequently, the final dataset comprised 214 samples, including 206 pup samples and 8 maternal samples.

### Protein extraction, protein digestion, and peptide clean-up

The metaproteomic workflow used here was previously benchmarked as a DIA-PASEF-based approach for host–microbiome analysis, showing increased proteome depth and improved detection of low-abundance host and microbial proteins compared with conventional workflows [29]. In the present study, we applied this established workflow to low-input fecal samples from early postnatal mice, including D10–D12 samples with very limited fecal material and low microbial biomass.

The sequence of the FS collected was randomized, and samples were relocated in new boxes and processed for analysis by a blinded operator.

The protein preparation protocol, as previously described [41], started by taking 30–50 mg of FS that was mixed with 200 μL of lysis buffer (5% SDS, 2 M urea, 50 mM Tris–HCl, protease inhibitor 1×) and vortexed vigorously for 1 min. The samples were then placed in a Thermomixer (serial number: 5382JR638726, Eppendorf, Hamburg, Germany) at 1200 rpm and 70 °C for 15 min. The samples were then ultrasonicated using a Bioruptor Pico (Diagenode, Seraing, Belgium, program: 15 cycles of 30 s “on” and 30 s “off,” frequency level: low, water temperature: + 20 °C). At room temperature, the tubes were then centrifuged for 5 min at 16,000 g. The supernatant was moved into a new tube, centrifuged again, and stored at −80 °C until protein digestion with SP3 was performed. In the case of early experimental time points (10–12 days) when the FSs range from 0.1 to 10 mg, all the sample collected was processed as reported above. A modified version of the one-pot solid-phase enhanced sample preparation method (SP3) of Hughes et al. [42] was used for protein purification and digestion was used. In brief, 50 μg of protein was added to a 1.5-mL LoBind tube (Eppendorf, Hamburg, Germany), and 50 μL of lysis buffer was added. Samples went through the processes of protein reduction (5 mM dithiothreitol, DTT, incubated for 30 min at + 60 °C) and alkylation (20 mM iodoacetamide, IAA, 30 min in the dark at room temperature). The remaining IAA in the sample was quenched by adding DTT at a final concentration of 5 mM. Premixed Sera-Mag SpeedBead beads were added in a volume of 10 μL (50 mg/mL, Cytiva, Marlborough, MA) to a 50 μg protein sample. One volume of absolute ethanol was immediately added to initiate the protein binding to the beads, followed by incubation and agitation at 1000 rpm for 5 min at 24 °C in a Thermomixer. After standing on a magnetic rack for 2 min, the supernatant was removed, and the beads were rinsed three times with 500 μL of 80% ethanol. The obtained beads were reconstituted in 50 μL of digestion buffer (50 mM ammonium bicarbonate, pH 8). Two micrograms of sequencing grade trypsin/LysC (Promega, Madison, USA) was used for protein digestion in a Thermomixer with agitation at 950 rpm for 18 h at 37 °C. After a 30-s spin, acetonitrile (ACN) was added to each sample to reach a final concentration of 95%. The mixture was incubated at room temperature for 8 min and then placed on a magnetic rack for 2 min. After the supernatant was discarded 900 μL of 100% ACN was added, 15 s on a magnetic rack and removed from each tube. To elute the peptides, the washed beads were reconstituted in 20 μL of liquid chromatography-mass spectrometry (LC–MS) grade water. Peptide concentrations were measured in duplicate at 205 nm using a NanoPhotometer N60 (serial number: TG2022, Implen, Munich, Germany). A final concentration of 0.1% Formic acid (FA) was added to acidify peptides and the tubes were stored at −20 °C until LC–MS/MS analysis.

### Liquid chromatography mass spectrometry (LC–MS/MS)

Nanoflow reversed-phase liquid chromatography (Nano-RPLC) was conducted using a NanoElute system (Bruker Daltonik, Bremen, Germany). A total of 250 ng of digested peptides were loaded onto a trap column (Neo-Trap C18, 5 mm × 300 µm, Thermo Fischer) and then separated on a 25 cm × 75 µm column, packed with 1.7 µm C18 particles (IonOpticks, Fitzroy, Australia), using a 60 min gradient. Mobile phase A consisted of 100% water (LC–MS grade) with 0.1% formic acid (FA), while mobile phase B was 100% acetonitrile with 0.1% FA. The separation was performed at a flow rate of 250 nL/min, except for the final 7 min, during which the flow rate was increased to 400 nL/min. The gradients included a linear increase of mobile phase B from 4 to 20% over the first 35 min, followed by an increase to 35% over 17 min. Subsequently, mobile phase B was ramped up to 85% within 0.5 min and held at this level for 7 min to elute hydrophobic peptides. Peptide eluates were analyzed on a hybrid TIMS quadrupole TOF mass spectrometer (timsTOF Pro2, Bruker Daltonik) coupled via a CaptiveSpray ion source. Data acquisition was performed in data-independent acquisition (DIA) mode, in combination with parallel accumulation serial fragmentation (PASEF). The TIMS analyzer operated at 100% duty cycle with an accumulation and ramp time of 100 ms. Ion mobility separation was set in the range of 0.65–1.40 (1/k0). Precursors within an *m/z* range of 400–1200 were targeted in 12 scan cycles with 3 isolation steps in each cycle, comprising 32 isolation windows of 25 Th, resulting in a total cycle time of 1.38 s. Collision energy was ramped linearly from 65 eV at 1/k0 = 1.6 to 20 eV at 1/k0 = 0.6.

### Microbial database construction

The database used here was reduced based on fractionated samples of pooled mouse fecal peptides (in-house). The fractionated samples were acquired on both timsTOF Ultra and timsTOF Pro in DDA-PASEF mode. High pH reversed-phase fractionation of pooled peptides. Peptide fractionation kit was purchased from Fisher Scientific (Cat. 84,868). A total of 40 µg pooled peptides were processed according to the manufacturer’s instructions. Eight peptide fractions were dried using vacuum centrifugation and then re-suspended in 30 µL of MS-grade water. The peptide concentration was measured in duplicate using NanoPhotometer N60 (Implen, München, Deutschland) at 205 nm. Peptide samples were acidified with FA to a final concentration of 0.1% and stored at −20 °C until LC–MS/MS analysis. Data dependent acquisition (DDA-PASEF) of fractionated peptides on timsTOF Ultra and timsTOF Pro 20 µg of each peptide fraction was loaded on an AuroraTM ULTIMATE column (25 cm × 75 µm) packed with 1.6 µm C18 particles (IonOpticks, Fitzroy, Australia) with a total 66 min gradient. The mobile phase B was linearly increased from 5 to 23% in 56 min with a flow rate of 0.25 µL/min, followed by another linear increase to 35% within 4 min and a steep increase to 90% in 1 min. The mobile phase B was maintained at 90% for the last 5 min with a flow rate increase from 0.25 to 0.35 µL/min. On both timsTOF Ultra and timsTOF Pro, the TIMS analyzer was operated in a 100% duty cycle with equal accumulation and ramp times of 166 ms each. Specifically, 5 PASEF scans were set per acquisition cycle (cycle time of 1.03 s) with an ion mobility range from 0.7 to 1.3 (1/k0). The target intensity and intensity threshold were set to 14,000 and 500, respectively. Dynamic exclusion was applied for 0.4 min. Ions with m/z between 100 and 1700 were recorded in the mass spectrum. Collision energies were dependent on ion mobility values with a linear increase in collision energy from 1/K0 = 0.6 Vs/cm^2^ at 20 eV to 1/K0 = 1.6 Vs/cm^2^ at 59 eV. In addition, 100 µg of each peptide fraction was analyzed in a 90-min gradient on timsTOF Pro. In this setup, the mobile phase B was linearly increased from 0 to 20% in 65 min with a flow rate of 0.25 µL/min, followed by another linear increase to 35% within 15 min and a steep increase to 90% in 0.5 min. The mobile phase B was maintained at 90% until the end of analysis with a flow rate of 0.4 µL/min. The TIMS analyzer was operated in 100% duty cycle with 100 ms defined for accumulation and ramp time. Ten PASEF scans were set per acquisition cycle (cycle time of 1.17 s) with ion mobility range from 0.65 to 1.45 (1/k0). The target intensity and intensity threshold were set to 10,000 and 1750, respectively. DDA-PASEF data processing: three sets of fractionated data generated using timsTOF Ultra and timsTOF Pro were separately submitted to MSfragger (Version 3.7) integrated in the FragPipe computational platform (Version 19.1), searching against a metagenome-translated protein database (PD1; https://doi.org/10.1038/nbt.3353) containing 2.6 million protein sequences. The decoy database was generated with reversed sequences. Trypsin was specified with a maximum of 2 missed cleavages allowed. The search included variable modifications of methionine oxidation and N-terminal acetylation and a fixed modification of carbamidomethyl on cysteine. The mass tolerances of 20 ppm for both precursor and fragment were used.

Peptide length was set from 7 to 50 amino acids with a mass range from 500 to 5000 Da. The rest of the parameters were kept as default settings. During the validation, MSBooster (Version 1.1.10) was used for rescoring, and Percolator (version 3.5, default parameters) was checked for PSM validation. FDR level was set to 1% for PSM, peptide, and protein. The identified proteins from the search were combined to form a sample-specific protein database (PD2) containing 48,970 sequences.

### DIA-PASEF data processing

To process DIA-PASEF data [41], DIA-NN (Linux version 1.8.1) was used in library-free mode and based on a microbial database of 48,970 protein sequences and the standard Uniprot proteome of *Mus musculus* (UP000000589 *Mus musculus* reference proteome, accessed on 20230427). Within DIA-NN, a deep learning-based method was used to predict theoretical peptide spectra, retention time, and ion mobility. Trypsin/P was used for in silico digestion, with a maximum of 2 missed cleavages. Peptide identification allowed up to two variable modifications per peptide, including methionine oxidation and N-terminal acetylation. Carbamidomethylation on cysteine was selected as a fixed modification. Peptides were restricted to lengths between 7 and 50 amino acids. The *m/z* range was defined according to the DIA-PASEF acquisition method: 400–1200 for precursor ions and 100–1700 for fragment ions. Mass accuracy for both MS1 and MS2 was set to automatic determination. Protein inference was performed at the protein name level, with the “Heuristic protein inference” option disabled. Raw data files were processed in parallel. DIA-NN first searched each file individually against the predicted spectra library to generate a quant file. In the second step, DIA-NN built an empirical spectral library based on all quant files [43]. And lastly, all files were reprocessed against the empirical spectral library using the match-between-runs (MBR) feature. Quantification was carried out using retention time–dependent cross-run normalization with the “Robust LC (high precision)” setting.

The resultant precursor intensities were further processed using the R package DIA-NN (v1.0.1; https://github.com/vdemichev/diann-rpackage) to extract and compute the MaxLFQ, quantitative intensity for all detected peptides and protein groups with *q*-value < 0.01[44].

### Microbiota taxonomy, function, functional redundancy, and taxa-function analyses

The built-in taxonomy and function databases were created by importing into iMetaLab (v2.3.0); peptide and protein tables were generated with MaxLFQ[45]. Tables with peptide and intensities data (microbial and host) were adopted for taxonomical annotation (Ignore blanks under rank: Superkingdom. Unique peptide count ≥ 3, with Peptide-Spectrum Match FDR equal to 0.01), while protein identifications with corresponding intensities were adopted for functional annotation (with default parameters).

The taxonomy annotation output from iMetalab was further processed in R by removing peptides without taxonomic annotations and taxa annotated by fewer than three distinct peptides across the full dataset (Supplementary Table 1). Among the 17,133 peptides of the dataset, each peptide is only assigned to one taxon lineage with the LCA algorithm. Log2 intensities of peptides common to the same taxa were summed for each sample. Alpha and Beta diversity indexes, engraftment analyses, and taxa-function were calculated at the Species level. The relative abundance of a species was defined as the percentage of the peptide summed intensity average for each sex in each cohort. Analyses performed at the cage level are based on the cage shared by the mothers and their respective pups before weaning. Mothers’ Core Species (Species detected in at least 3 Mothers of the same cohort), and the Pups’ Core Species (Species detected in at least 3 pups of the same cages) were considered to assess the microbiota engraftment in pups of the same cohort over time. Oxygen tolerance (facultative versus obligate anaerobic metabolism) was assigned to bacterial taxa using curated trait information from publicly available microbial phenotype databases integrated in R, including BacDive-derived data and associated trait resources. Community abundances were then aggregated by oxygen category across samples and time points to quantify their relative contributions (https://github.com/scottdaniel/bacphene).

From the iMetaLab function annotation table, the KEGG pathways identified by fewer than 3 proteins across the full dataset were removed (Supplementary Table 2), recognizing that while this may reduce sensitivity to low-abundance or transient biological functions, it helps minimize stochastic detection and annotation-related noise. Since this filter was applied across the full dataset rather than within individual samples or time points, pathways transiently detected during early life could still be retained if they were supported by at least three proteins overall. Proteome-level functional redundancy (FRp) was calculated as described by Li and colleagues [46]. Briefly, FRp estimates the degree to which expressed microbial functions are shared across multiple detected taxa, based on taxon-function assignments derived from metaproteomic data. We used the normalized FRp (nFRp) described by Li et al. to account for differences in the number of detected taxa and functions across samples, enabling comparison of functional redundancy across developmental stages. Biologically, higher nFRp indicates that a larger proportion of expressed functions are represented by multiple taxa, whereas lower nFRp indicates that functions are more taxon-restricted. Data preparation was performed using Python 3.12.6 and PhyCharm Community Edition 2024.2.3. To define the Species-specific functions Meta4P [47] was used by importing the tables with the peptide intensities for each microbial in each sample (Supplementary Table 1) and the taxonomic and functional annotation tables (Supplementary Table 2). Meta4P results were filtered by the Species-NOG pathway identified by at least 3 peptides.

The log2-transformed intensities of common peptides annotated in the same protein were summed across each sample. Consensus clustering analysis was adopted to evaluate the stability of KEGG pathway clustering and determine the optimal number of clusters (k). Analyses were conducted separately for each mouse cohort with pooled sexes using z-score–transformed KEGG pathway expression profiles. Consensus clustering was performed for k values ranging from 2 to 6 using 500 bootstrap resampling iterations, with each iteration subsampling 80% of the samples and 80% of the features without replacement. Hierarchical clustering based on Euclidean distance and Ward linkage was used for cluster assignment. Silhouette scores and consensus stability metrics were used to identify the optimal number of clusters. Consensus clustering was selected over Calinski–Harabasz (CH), Davies–Bouldin (DB), and Gap statistic approaches to provide a more robust resampling-based estimate of cluster stability. All analyses were performed in Python using numpy, pandas, scikit-learn, and seaborn.

### Host proteome analysis

The protein intensity, from the DIA-NN host protein sequences, was summed among common peptides in the same sample and divided by the total intensity of the sample (Protein Relative Abundance). Considering the factors time and cohort soft clustering (allowing proteins to belong to multiple clusters) with the Mfuzz R package was performed [48]. First, data were pre-processed by averaging replicates at each condition and standardizing each protein’s profile (z-scoring). By examining the Mfuzz Dmin plot, the number of clusters at the Dmin inflection point (the “elbow point”—where the minimum centroid distance stops decreasing rapidly and begins to level off) was used for the optimal number of cluster definition. With the estimated fuzzifier (m≈1.25, consistent with low–moderate noise structure), data-driven estimation avoided arbitrary selection of fuzzification and ensured appropriate membership smoothing. The optimal number of clusters was determined using a combination of (i) the Dmin function (minimum centroid distance) and identification of the elbow inflection point, and (ii) comparison with the within-cluster sum of squares (WSS) elbow from k-means clustering as an orthogonal criterion. Both approaches converged on a consistent range of cluster numbers across cohorts. Cluster robustness was evaluated using consensus clustering based on repeated subsampling (100 iterations). For each iteration, Mfuzz clustering was performed, and proteins were assigned according to maximal membership values. Consensus matrices representing the frequency of co-clustering between protein pairs were used to derive cluster stability scores [48]. The plots of the mean profile for each cluster were generated considering proteins with the highest membership score (> 0.7–0.9) to each cluster detected. Proteins with the highest membership were searched for Multiple Proteins by Names / Identifiers on String 12.0 (https://stringdb.org; 23/05/2025), and protein full STRING networks were obtained with confidence as a parameter for the meaning of the network edges, with a minimum required interaction score of 0.7 (high confidence). The host proteins were matched by Peptide sequence or Protein Name to the 152 known antimicrobial peptides (AMP) reported by Valdés-Mas and colleagues [49]. AMPs were selected for focused analysis due to their central role in innate immune defense and their established function in shaping gut microbial composition. Valdés-Mas and colleagues [49] showed that host-derived AMPs are dynamically regulated over time and can co-vary with inflammatory host proteins in an intestinal inflammatory disease mouse model. This supports their use as sensitive markers of host–microbiome immune interactions and temporal immune modulation in longitudinal datasets. The protein intensity was summed among common peptides in the same sample and divided by the total intensity of the sample (relative AMPs abundance). Due to the high number of low-abundant AMPs, a reduced dataset considering the 10 most abundant AMPs among all the samples was adopted to perform analyses of the main phenomena of the AMPs evolution over time and to detect differences between pups of different mothers’ origin and sexes. Finally, the carbohydrate-active enzymes (CAZymes) were selected for targeted analysis because of their key role in regulating the structural and nutritional landscape of the gut environment. CAZymes were explored for their involvement in the synthesis, modification, and degradation of carbohydrates, thereby influencing microbial access to host-derived glycans, epithelial barrier properties, and microbial colonization dynamics, especially during the weaning transition [50–52]. To our knowledge, no previous work on a proteomic level has addressed the presence and role of CAZymes during mouse development, suggesting an underexplored area in developmental host–microbiome research. The dataset was created by selecting among all host proteins those annotated on UniProt (search performed on 28/02/2025) matching the CAZymes categories listed by Wardman and colleagues [50]. To explore host–microbe transitions in glycan metabolism, microbial CAZymes from the iMetaLab function annotation table were grouped into functional categories, including host glycan/mucin degradation, plant glycan degradation, pectin degradation, starch/glycogen metabolism, chitin/cell wall degradation, glycosaminoglycan degradation, and glycan biosynthesis [53–57]. The microbial CAZymes temporal abundance patterns were evaluated across developmental stages.

### Food proteome analysis

The food protein dataset was generated using the FASTA file created by Valdés-Mas and colleagues [49] through FragPipe (https://fragpipe.nesvilab.org/) to generate a reduced protein list. An independent database, separate from the host and microbiome protein datasets, was generated for dietary protein analyses. As previously reported by Valdés-Mas et al., broad database searches can lead to over-detection of food proteins because of varying degrees of sequence identity and alignment coverage among homologous proteins. To minimize this issue, the interpretation database was restricted to proteins originating from plant genera corresponding to the species listed in the mouse chow ingredients (Triticum, Glycine, Zea, Avena, and Beta) together with mouse milk proteins downloaded from UniProt (search performed on 28.02.2025; Supplementary Table 3 contains all dietary protein accessions). Only unique protein markers for each genus were retained for downstream analyses to avoid detection bias. “Unique protein markers” were defined as proteins specifically associated with the curated food or milk sources and not shared with homologous host proteins; therefore, ambiguous proteins lacking dietary-source specificity were excluded from the food protein dataset. Lactoferrin is a secreted protein present in the milk and the intestinal mucosa [58, 59]; for this reason, it was differentiated from the other milk proteins in the analyses. For relative quantification, protein intensities were summed across common peptides within the same sample and normalized to the total peptide intensity of the sample, in order to obtain an unbiased estimate of relative food protein abundance.

The final curated database included a total of 211 proteins, comprising 92 Triticum, 37 Glycine, 72 Zea, 3 Avena, 2 Beta, and 5 milk proteins.

### Statistical analyses

The lists of Species detected in pups and mothers were compared with those reported in the literature as core shared species between C57BL/6 J mice and humans [27, 28].

Gut microbiota composition statistical analysis was performed based on mixed-effects models because they appropriately accommodate unbalanced longitudinal sampling and missing observations across time points. Individuals with incomplete sampling were retained in the analysis, and all available observations contributed to model estimation through maximum likelihood procedures. No imputation of missing values was performed. Generalized linear mixed-effects models or linear-mixed effects models were adopted to account for the hierarchical structure of the experimental design and repeated measures over time. Specifically, samples collected longitudinally across developmental time points were nested within shared experimental units, including litter, maternal identity, and cage. We evaluated the contribution of each hierarchical level by examining the variance components of the random effects. This procedure allowed us to assess the extent of pseudoreplication arising from shared pups, experimental units, maternal origin, litter membership, and cage housing. Final model specification was guided by both model convergence and the empirical contribution of each random effect to variance explanation, resulting in a parsimonious structure retaining only those levels that contributed non-negligible variance, avoiding overparameterization and convergence issues. Estimated marginal means with BH correction to perform the post hoc analyses of the significant interactions between the factors time, sex, and cohort if they were adopted. The α-diversity (Richness Shannon, and Simpson indexes) and β-diversity metrics (Bray–Curtis and Jaccard dissimilarities) were calculated by using vegan (version 2.7-3packages in R 4.6.0 (R Core Team, 2018). Statistical analyses for the α-diversity indexes were performed with generalized linear mixed-effects models based on the criteria described above.

For β-diversity indexes, random seed initialization was fixed (using set.seed (12345)) to ensure reproducibility of permutation-based analyses, and PERMDISP results were reported alongside PERMANOVA to assess homogeneity of multivariate dispersion. The Benjamini–Hochberg (BH) correction for pairwise multilevel comparisons under a reduced model were employed to detect the relative contributions to microbial community variability from time, sex, and cohort. The same strategy was adopted to compare the metabolic-informed taxonomical index between the pups and their mothers. The analyses of the data from fecal samples were performed with rstatix_0.7.3, ggplot2_4.0.3, ggpubr_0.6.3, lme4_2.0–1, emmeans_2.0.3, stringr_1.6.0, ggVennDiagram_1.5.7, purrr_1.2.2, vegan_2.7–3, pairwiseAdonis_0.4.1.cluster_2.1.8.2, lme4_2.0–1, Matrix_1.7–5, emmeans_2.0.3, and nlme_3.1–169 R packages. Body weight measurements were recorded before the FS collection (Sartorius LC621P) and are reported in Supplementary Table 18. Although body weight was considered as a potential explanatory variable, it was not included in the final mixed-effects models because it was strongly correlated with age (time point), one of the principal factors of interest in the study (Spearman’s rank correlation, *ρ* = 0.873, *p* < 2.2 × 10⁻^1^⁶). The simultaneous inclusion of both variables would therefore introduce substantial collinearity into the analyses. Sex-specific body weight differences emerged from postnatal day 20 onward, while no significant differences were observed between the two cohorts (Supplementary Table 18). All data are represented as mean ± SD (standard deviation of the mean) unless indicated otherwise. All replicates are biological unless indicated otherwise. All statistical tests are reported in the respective figure caption. Significant interactions for taxonomical and functional analyses were assessed for the normality of the data distribution. In all panels: *p* > 0.05; *p < 0.05; **p < 0.01; ***p < 0.001; ****p < 0.0001.

## Results

### Unveiling taxonomic developmental programs during early life

**Fig. 1:**
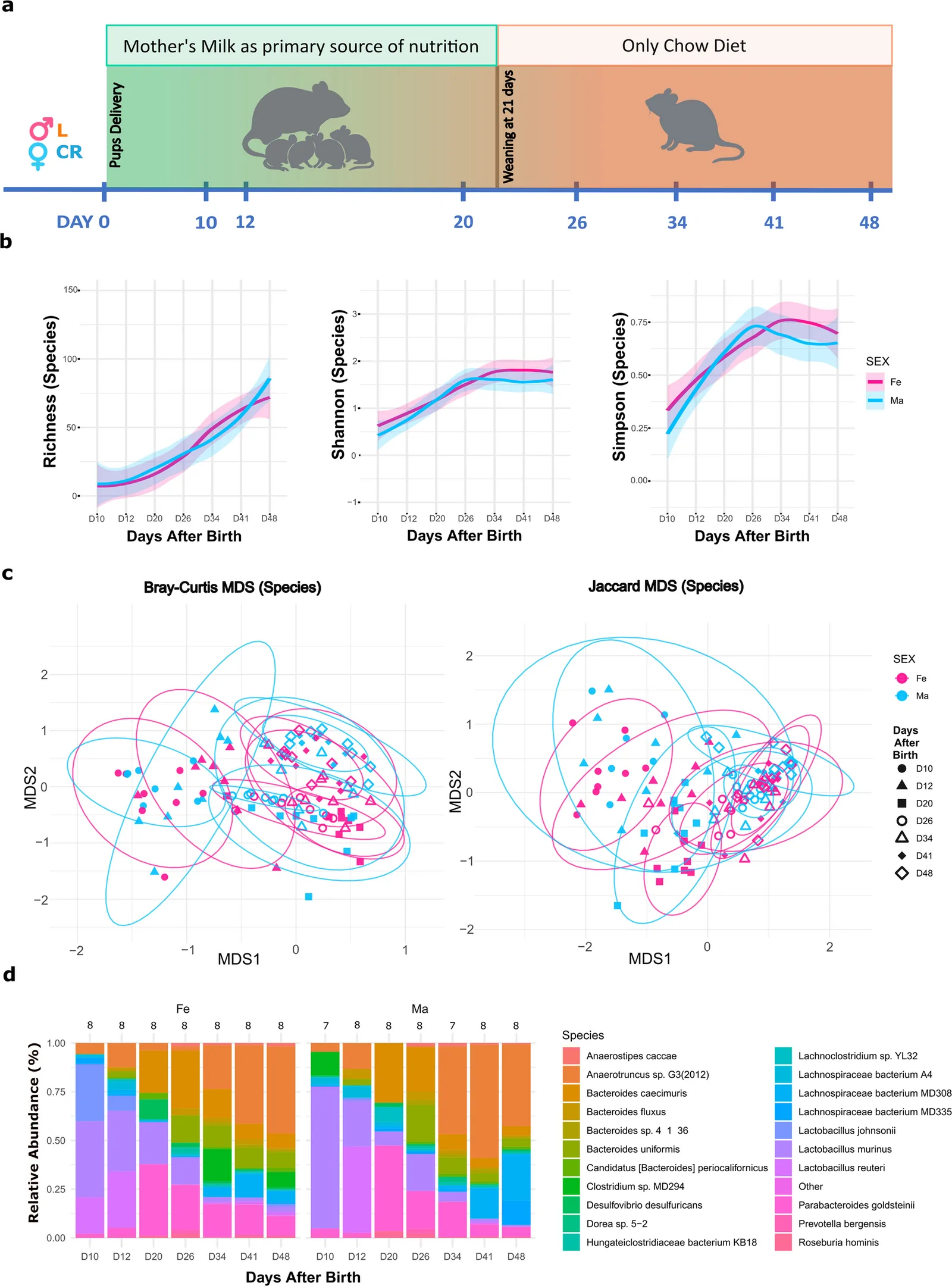
Taxonomic maturation of gut microbiota in local pups. **a** Experimental design to characterize the microbiota composition of 16 pups (eight females and eight males) for each cohort (local, L, and Charles River, CR) is shown schematically. Before weaning, pups were naturally fed by their moms with milk ad libitum. However, chow was provided in the cage for the mother’s feeding. After weaning on day 21, the pups were caged and divided by sex with siblings from the same mother for 27 days and fed exclusively with chow. The microbiota from pups’ fecal samples was examined on days 10, 12, 20, 26, 34, 41, and 48 after birth. **b** Alpha diversity indices (Richness, Shannon, Simpson) were calculated at the species level at each time point. Data are shown as mean (thick line) ± 95% confidence intervals, with sexes indicated by different colors. As data were not normally distributed, mixed-effects models with Benjamini–Hochberg correction for multiple testing were applied. All alpha diversity indices showed a significant effect of Time (Richness: *χ*^2^(1,6) = 164,58, *p* < 0.0001; Shannon: *χ*^2^(1,6) = 93,98, *p* < 0.0001; Simpson: *χ*^2^(1,6) = 77,38, *p* < 0.0001). **c** Non-metric multidimensional scaling plots based on Bray–Curtis dissimilarity and Jaccard indices illustrate beta diversity at each time point (stress = 0.19; 999 iterations). Permutation test for Adonis (PERMANOVA) with Benjamin–Hoch correction for pairwise multilevel comparison under reduced model revealed for Bray–Curtis index a significant overall effect of Time F(1,6) = 13,26; *p* < 0.001. Also, for the Jaccard index, a significant overall effect of Time F(1;6) = 8,29; *p* < 0.001 was detected. **d** Relative Species abundance is reported as the percentage of the mean peptides’ summed intensity for each Species among the pups of the same sex (Fe = female, Ma = male). Species detected with relative abundance below 1% had been reported as “Other.” Above each column of the histogram is reported the number of mice of the same sex analyzed

To characterize the temporal assembly of the gut microbiota during early life, we performed longitudinal metaproteomic profiling of fecal samples collected from postnatal day (D) 10, the earliest time point at which fecal material was available, through D48 (Fig. 1a). Samples were obtained from male and female pups belonging to two C57BL/6 cohorts that differed only in maternal origin: pups born to a long-established local breeding colony (L) and pups born to pregnant females newly acquired from the same vendor (Charles River; CR). Importantly, both cohorts were born contemporaneously and raised under identical housing and dietary conditions (see Methods), allowing us to assess developmental trajectories of gut microbiota maturation while minimizing environmental confounders and acknowledging any potential genetic drift due to inbred local mice.

Across both cohorts and sexes, gut microbial α-diversity increased markedly with age (Figs. 1b and S1a; Supplementary Tables 4–6; Mixed-effects models, all *p* < 0.0001). Species richness increased steadily throughout early development, whereas Shannon and Simpson diversity indices rose steeply during the first three postnatal weeks and then largely plateaued after D26. This pattern is consistent with the rapid establishment of dominant community structure and evenness during the pre-weaning period, followed by continued accumulation of low-abundance taxa over time after dietary transition to solid food. No significant differences in α-diversity were detected between male and female pups within the same cohort at any time point (Supplementary Tables 4–6).

Temporal restructuring of the microbial community was further supported by β-diversity analyses. Bray–Curtis and Jaccard dissimilarities showed similar developmental separation patterns. They both revealed significant separation of samples by developmental stage in both cohorts (PERMANOVA, *p* < 0.001; Figs. 1c and S1b, Supplementary Tables 8–9), with early-life communities at D10–D12 clustering distinctly from those observed after weaning (D34–D48). Both these indices revealed a significant difference between the two cohorts from D20 to D34 and at D48, independently of the sex of the pups (PERMANOVA Time × Cohort; *p* < 0.001; Supplementary Table 7–8). These age-dependent shifts were consistent across sexes, indicating reproducible changes in gut community structure during development.

Beyond ecological indices, metaproteomic profiling enabled species-resolved taxonomic analysis of microbial turnover across development. The number of confidently detected species increased progressively across development, from 36 species at D10 to 198 species at D48 (Supplementary Table 9), consistent with the expected maturation and increasing taxonomic complexity of the gut microbiota over time, following similar trajectories across cohorts and sexes (Figs. 1d and Supplementary 1c), with detectable individual heterogeneity (Supplementary Fig. 2). Early-life samples were dominated by facultative anaerobes (~ 31–46%), primarily Lactobacillus species, including *L. murinus*, *L. reuteri*, and *L. johnsonii*. As development progressed, these taxa were progressively replaced by obligate anaerobes (20–30%) such as *Anaerotruncus* sp. G3 (2012), *Bacteroides caecimuris*, and members of the Lachnospiraceae family (Supplementary Table 9). This taxonomic succession was concomitant with the weaning period and the transition from maternal milk to solid chow, consistent with the establishment of a more complex gut ecosystem during postnatal maturation.

**Fig. 2:**
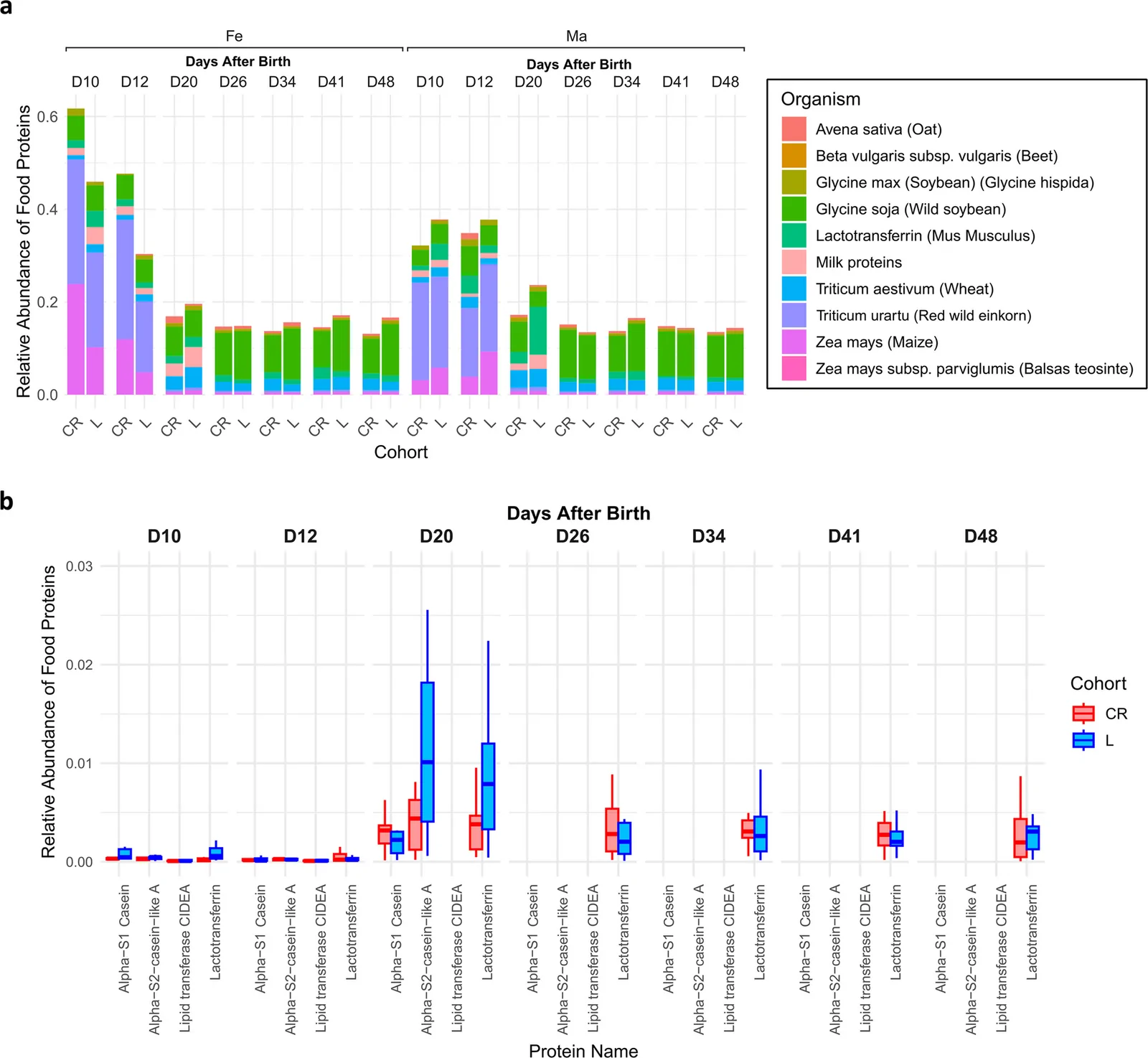
Detection of food proteins detected during the postnatal development. **a** All proteins are presented as mean normalized relative abundances across the same category (time, cohort, sex). The mean normalized relative abundance was calculated as the summed peptide intensity for each protein divided by the total peptide intensity of the sample. Estimated marginal means with Benjamin–Hochberg correction from Generalized Mixed-Effects Models including the fixed effects of Time, Cohort, SEX, and all their interactions (Time:Cohort:SEX), while accounting for repeated measurements within individuals (ID) and shared post-weaning housing conditions (cage_post) as random intercept effects. The analysis revealed a significant effect of developmental time on Triticum urartu (Type III Wald *χ*^2^(6) = 355,86, *p* < 0.0001) and Zea mays (*χ*^2^(6) = 129,98, *p* < 0.0001), with significantly higher abundance in the early timepoints. **b** Mean normalized relative abundances of the maternal milk proteins between local and CR pups throughout development; *n* = 16 pups per cohort and time point (8 females and 8 males)

### Dynamics of dietary protein during early life

Because dietary inputs are a primary driver of gut microbiota assembly dynamics during early life [60], we next quantified food-derived proteins in pup fecal samples to pinpoint the transition from maternal milk to solid chow. Although dietary proteins accounted for only a small fraction of the total fecal proteome, we reproducibly detected them across developmental stages, which enables direct assessment of dietary transitions in vivo and underscores the sensitivity of our metaproteomic method.

Across both cohorts and sexes, we revealed a marked and reproducible shift in dietary protein profiles over time (Fig. 2a). After weaning, chow-associated proteins derived from *Zea mays* and *Triticum urartu* decreased significantly in detectability (glmem, Time; *p* < 0.0001; Supplementary Table 10), while soybean- and wheat-derived proteins showed relatively constant detectability across timepoints. Proteins originating from maternal milk were detected exclusively during the pre-weaning period. Among milk-associated proteins, α-caseins were consistently detected through D20 but were absent thereafter, reflecting the cessation of milk intake around weaning (Fig. 2b). In contrast, Lipid Transferase CIDEA showed low abundance at D10–D12 and was no longer detectable by D20, suggesting early functional changes in lipid processing concurrent with microbial and dietary maturation. Notably, lactotransferrin was detected across the entire developmental window in both cohorts (Fig. 2b; Supplementary Table 10). This persistence likely reflects a transition in the source of lactotransferrin, from maternal milk before weaning to endogenous secretion by the intestinal mucosa after weaning [58, 59]. Indicative of a complete transition to solid food, main chow-derived components, like soybeans and wheat, that were constantly detected, and *Zea mays* and *Triticum urartu* progressively decreased after weaning.

### Functional specialization underlies increasing microbiome functional redundancy

**Fig. 3:**
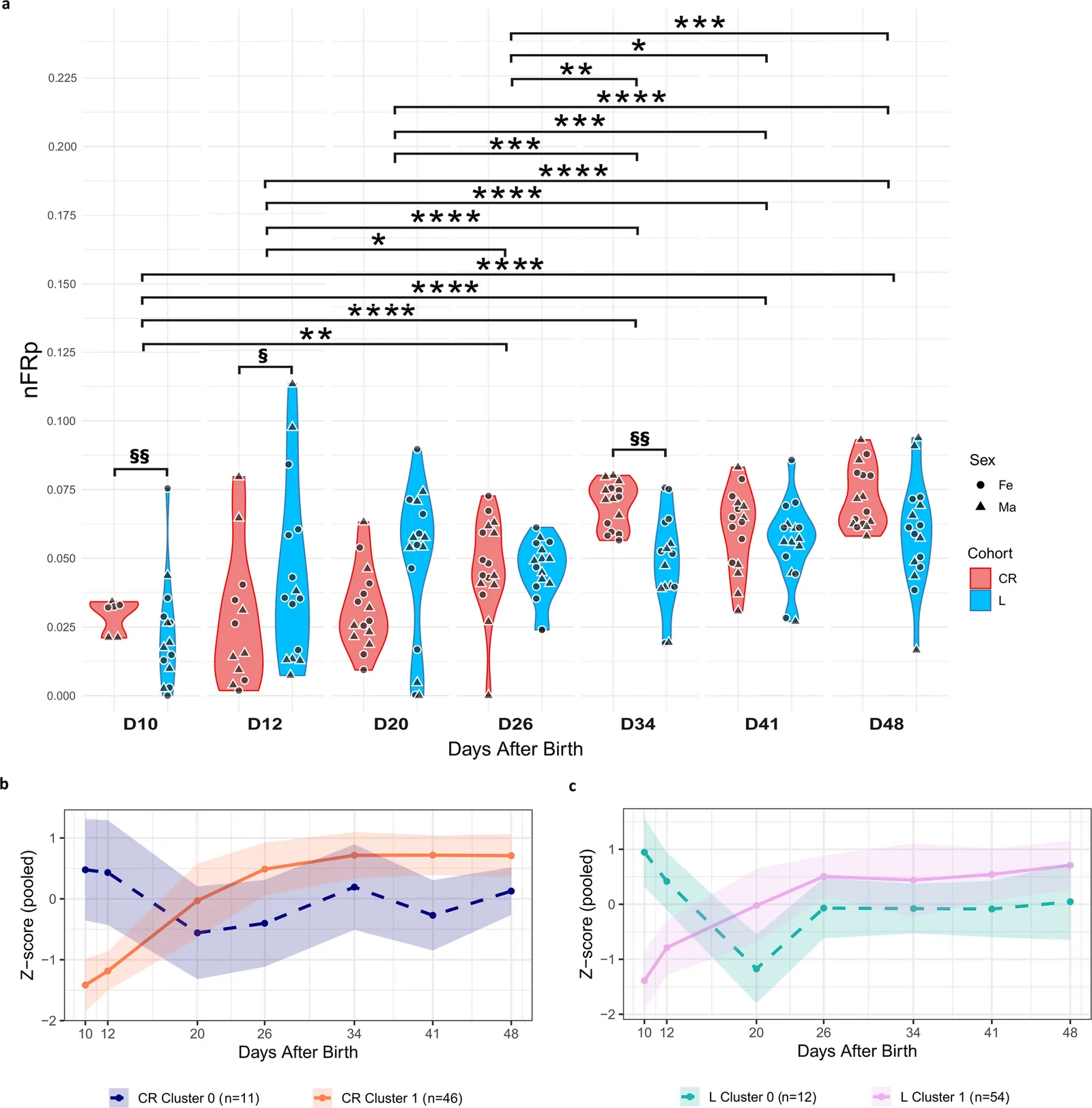
Functional redundancy and gut microbiota functional clustering. **a** Normalized functional redundancy index of the proteome (nFRp) values for pups of different cohorts is reported. Estimated marginal means with Benjamin–Hochberg correction from Linear Mixed-Effects Models were adopted. The model included the fixed effects of Time, Cohort, SEX, and all their interactions (Time * Cohort * SEX), while accounting for repeated measurements within individuals (ID) and shared post-weaning housing conditions (cage_post) as random intercept effects. The analysis revealed a significant effect of developmental time on nFRp (Type III Wald *χ*^2^(6) = 43.15, *p* < 0.001) and a significant Time × Cohort interaction (*χ*^2^(6) = 15.68, *p* < 0.016), indicating that developmental trajectories differed between cohorts. No significant main effects of sex or cohort were observed, and the three-way interaction between Time, Cohort, and Sex was not significant. *n* = 32; 16 for each experimental group (8 females and 8 males). Each point in the violin plot represents a pup’s sample. Differences between different time points are reported. * *p* < 0.1, ** *p* < 0.01,*** *p* < 0.0001, and **** *p* < 0.00001. Differences between cohorts over time are reported ^§^ *p* < 0.1, and ^§§^ *p* < 0.01. **b** Temporal clustering of microbial metabolic pathways in the CR cohort. Pathways were clustered based on their standardized expression (Z-score) profiles across time. Two distinct temporal patterns emerged: Cluster 0 (blue, dashed line, *n* = 11 pathways) and Cluster 1 (orange, solid line, *n* = 46 pathways). Lines represent cluster means, while shaded areas indicate the standard deviation of the mean (SD). **c** Temporal clustering of microbial metabolic pathways in the L cohort. Pathways were clustered based on their standardized expression (Z-score) profiles across time. Two distinct temporal patterns emerged: Cluster 0 (green, dashed line, *n* = 12 pathways) and Cluster 1 (pink, solid line, *n* = 54 pathways). Lines represent cluster means, while shaded areas indicate the standard deviation of the mean (SD)

To determine how age-dependent increases in taxonomic complexity and concurrent dietary transitions translate into functional organization of the gut microbiome, we quantified ecological functional redundancy using the normalized functional redundancy index of the proteome (nFRp). In both mouse cohorts, nFRp increased significantly over time, reaching a maximum around postnatal day 34 (Fig. 3a; lmem accounting for repeated measurements within individual, and shared post-weaning housing condition, Time; p < 0.0001; Supplementary Table 11). Although transient differences between cohorts were observed at intermediate time points, nFRp values converged by D41 (lmem, Time × Cohort; p < 0.016, Supplementary Table 11). No significant effect of the pups’ sex on the nFRp was detected over time and among the cohorts. This temporal pattern occurred alongside increasing species richness and taxonomic diversification, suggesting an association between community maturation and broader overlap in expressed functional capacity. However, these parallel trends do not establish a causal relationship between richness and functional redundancy. The total number of represented KEGG pathways increased from 105 on D10 to 140 on D48 (Supplementary Table 12). Consensus clustering of KEGG pathway expression profiles identified two major temporal patterns in each cohort (Fig. 3b and 3c), with no detectable sex-specific effects. Cluster 1, comprising approximately 80% of KEGG functions (Supplementary Table 13), showed progressive upregulation during early development followed by stabilization around D34, closely mirroring the nFRp trajectory in both cohorts. These functions encompassed pathways associated with carbohydrate and lipid metabolism, utilization of host-derived glycans, and stress-response-related processes that increased during development (Figs. 1d and 2a). In contrast, Cluster 0 showed a more modest change over time, characterized by a transient decrease around the weaning transition followed by partial recovery or stabilization at later time points. This cluster reflected functions related to core biosynthetic and housekeeping processes, such as peptidoglycan biosynthesis and amino acid metabolism.

### The origin of the mother is associated with taxonomic and functional engraftment trajectories

**Fig. 4:**
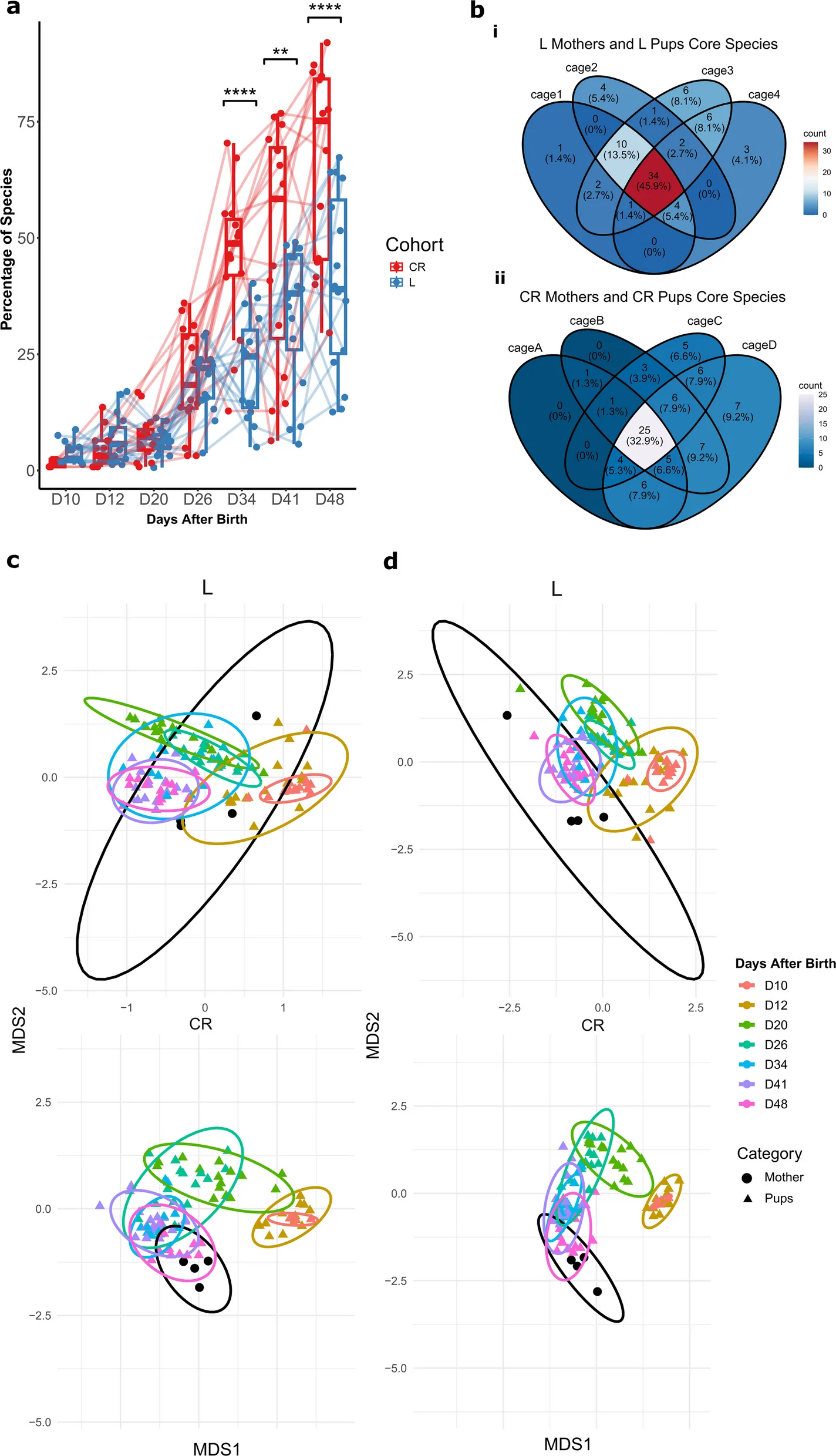
Engraftment of the mothers’ gut microbiota over time. **a** Engraftment of the two pups cohort over time. The plot reports the percentages of the mothers’ core species (detected in at least 3 Mothers of the same cohort) over time. Estimated marginal means with Benjamin–Hochberg correction from Linear Mixed-Effects Models detected a significant overall effect of Cohort F(1;6) = 14,33; *p* < 0.0007, and Time*Cohort F(1;6) = 7,38; *p* < 0.00001 (see Table S13). Differences between different time points are reported. ** *p* < 0.01, and **** *p* < 0.00001. *n* = 16 pups for each cohort and time point (8 females and 8 males). **b** Venn diagrams illustrates the relationship, in the local (i) and Charles River (ii) cohorts, between the mothers’ core species (species detected in at least 3 mothers) and the pups’ core species (species detected in at least 3 pups of the same cages) on day 26 after the delivery of the pups, categorized by the provenance cage. *n* = 20 mice (8 females and 8 males, and 4 mothers). **c** Non-metric multidimensional scaling (NMDS) plots based on Bray–Curtis β-diversity of metabolic-informed taxonomic profiles across pup developmental stages and cohorts. Within each cohort and pup developmental time point, maternal samples were compared with pups using Category (Mother versus Pup) as the PERMANOVA factor. NMDS ordination was performed with a final stress value of 0.244 after 999 iterations. The corresponding cohort- and time-specific PERMANOVA results are reported in Supplementary Table S15 (Time_Cohort_Category_Bray). **d** NMDS plots based on Jaccard β-diversity of metabolic-informed taxonomic profiles across pup developmental stages and cohorts. Within each cohort and pup developmental time point, maternal samples were compared with pups using Category (Mother versus Pup) as the PERMANOVA factor. NMDS ordination was performed with a final stress value of 0.165 after 999 iterations. The corresponding cohort- and time-specific PERMANOVA results are reported in Supplementary Table S15 (Time_Cohort_Category_Jacc)

Analysis of maternal fecal samples collected 26 days after delivery revealed substantial overlap in gut microbiota composition between L and CR mothers, with approximately 65% of maternal species shared (Supplementary Fig. 3a). Nonetheless, each maternal group harbored distinct subsets of taxa, with L mothers carrying more than one-quarter of core species not detected in CR dams. Maternal communities from CR and L dams were largely overlapping, with 112 shared species. L mothers contained 47 cohort-specific species, whereas CR mothers contained 13 cohort-specific species, indicating that the lower maternal-source contribution observed in L pups was not due to reduced maternal species detectability in L dams. Rather, this pattern suggests that cohort differences in offspring maternal-species overlap may reflect differences in acquisition, retention, or persistence of maternal taxa, as well as the specific identity of taxa available for transmission. Longitudinal tracking of maternal-detected species engraftment in offspring revealed a significant increase in maternal-associated species over developmental stages (Fig. 4a; Supplementary Table 14). This pattern was consistent with the age-dependent restructuring of microbial communities observed in β-diversity analyses (Fig. 1c and Supplementary Fig. 1b) and with the progressive functional maturation of the gut microbiome after weaning (Fig. 3a). From D34 onward, CR pups exhibited significantly higher levels of maternal species acquisition compared to L pups (Fig. 4a), suggesting cohort-associated differences in engraftment trajectories. These differences were independent of pup sex, as confirmed by sex-stratified analyses (Supplementary Fig. 3b). Analysis of pup core species—defined as taxa detected in at least three pups per cage at D26—revealed limited cage-specific effects, with most species shared across multiple maternal cages (Fig. 4b). Interestingly, a subset of species was shared across multiple, but not all, L and CR cages, 26 days after birth, suggesting variable engraftment dynamics. However, richness analyses showed that individual variability and the post-weaning housing cage contributed more strongly to species colonization variance than the cage of origin, while maternal-origin-associated effects remained detectable in cohort-specific taxonomic–functional trajectories during gut microbiome development.

To assess whether maternal origin was also associated with the development of taxonomic–functional structure, we compared metabolic-informed taxonomic profiles between mothers and their corresponding offspring. Since maternal samples were collected only at D26, maternal microbiota was not evaluated longitudinally. Instead, maternal profiles were used as a reference for comparison with pups at each developmental stage within each cohort. These comparisons were evaluated separately using Bray–Curtis and Jaccard PERMANOVA. β-diversity analyses based on Bray–Curtis and Jaccard dissimilarities indicated that pup microbiomes followed developmental trajectories progressively approaching maternal community configurations over time, remaining distinct only for the local cohort (Fig. 4c–d; Supplementary Table 15). Importantly, these community-level patterns do not directly reflect the proportion of maternally shared species shown in Fig. 4a, as Bray–Curtis and Jaccard metrics capture overall compositional structure and relative abundance relationships rather than simple taxonomic overlap alone. The pups from the two cohorts remained significantly different from their respective mothers and from their peers of the other cohort in taxonomic–functional composition across all time points from D12 to D48 (Supplementary Table 15), indicating that maternal complexity in mice is not reached at D48 and imprints cohort-specific functional trajectories during gut microbiome development.

### Maturation of the host intestinal proteome parallels the development of the gut microbiome in early life

To determine how microbiome maturation is mirrored by host intestinal physiology, we analyzed host-derived proteins quantified by metaproteomics across time points. In total, more than 3700 host proteins were detected across both mouse cohorts, of which 3699 were shared, and 60 and 70 were unique, respectively, in L and CR mice. Over the full developmental period, 1327 and 691 host proteins were significantly regulated in L and CR pups, respectively, whereas only 69 proteins showed differential temporal regulation between cohorts (lmem, Time × Cohort; *p* < 0.0001; Supplementary Table 16), indicating a largely conserved host developmental program. The pups’ sex over time did not affect the host proteins significantly, while 66 proteins were detected to be significantly different between same-sex peers of the two cohorts at a few time points at weaning and after (lmem, Time × Cohort × Sex; *p* < 0.0001; Supplementary Table 16).

**Fig. 5:**
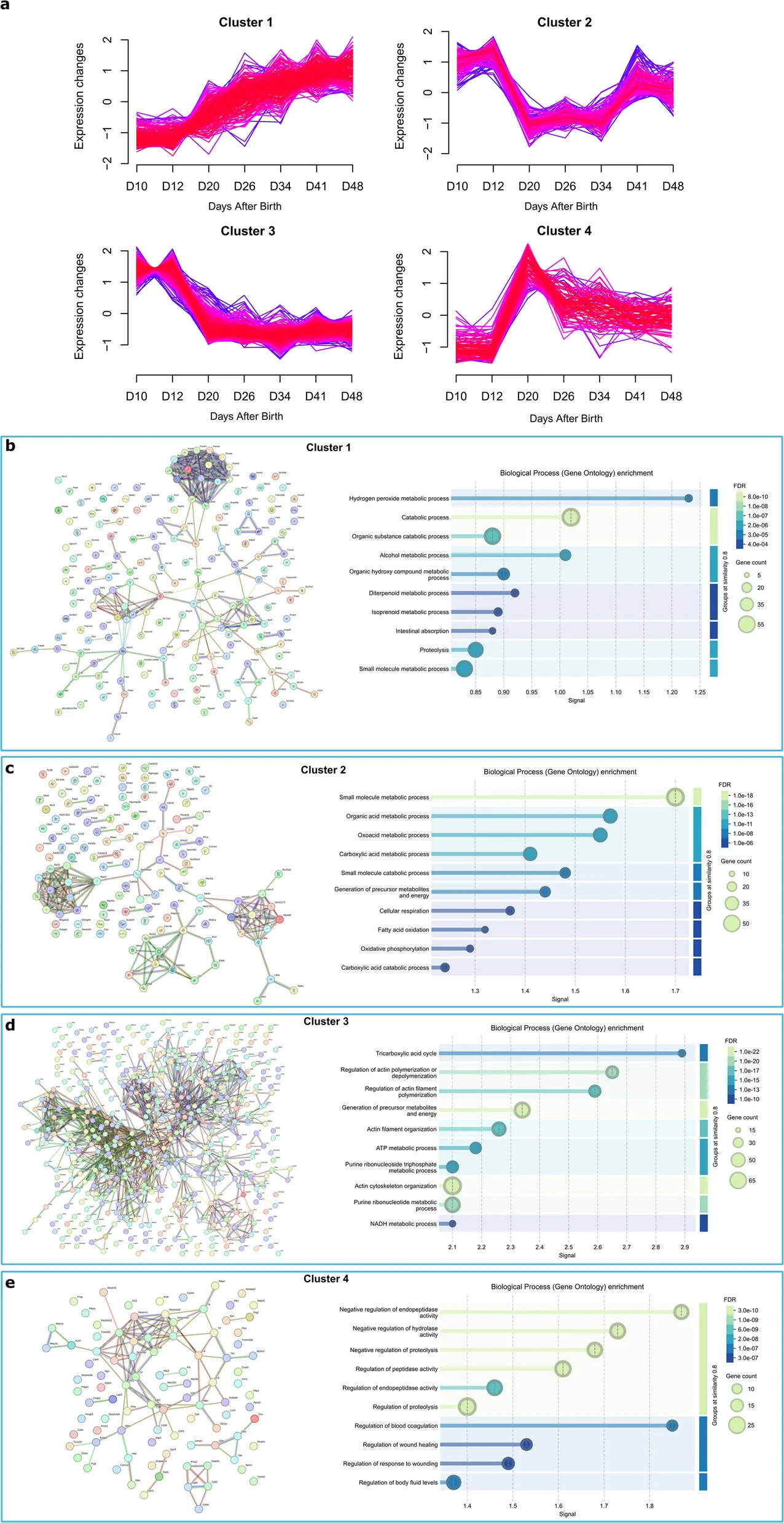
Temporal dynamics, clustering, and functional enrichment of host gut proteins in L pups. **a** Protein clusters are plotted as the mean standardized intensity, considering proteins with the highest membership score (> 0.7–0.9) in each detected cluster. Each line represents an individual protein, with color intensity reflecting membership strength (blue representing low and red representing high intensity). **b**–**e** Protein–protein interaction networks and Gene Ontology enrichment analyses for proteins with the highest membership score (> 0.7–0.9). Protein full STRING networks obtained with evidence as a parameter for the meaning of the network edges, with a minimum required interaction score of 0.7(high confidence), were generated using STRING12.0 (https://stringdb.org; 23/05/2025), and visualized with functional annotation. Clusters 1 (consensus stability = 0.981), 2 (consensus stability = 0.986), 3 (consensus stability = 0.997), and 4 (consensus stability = 0.996) are respectively composed of 190, 128, 492, and 96 proteins. Nodes represent proteins, and edges represent predicted interactions. The adjacent bar plots display the top enriched biological processes (GO terms) along with their corresponding gene counts and statistical significance (FDR). *n* = 16 mice (8 females and 8 males)

Cluster analysis of host protein expression profiles identified four major temporal patterns that captured the principal structure of the data (shown for L pups in Fig. 5a; CR pups shown in Supplementary Fig. 4). Consensus clustering analysis demonstrated high cluster robustness across repeated subsampling iterations (mean consensus stability ranging from 0.981 to 0.997 across clusters), supporting the reproducibility of the identified temporal trajectories. Protein–protein interaction network analysis and Gene Ontology enrichment revealed distinct functional trajectories across clusters of L pups (Fig. 5b–e), which were broadly recapitulated in CR pups (Supplementary Fig. 4).

Cluster 1 comprised 190 proteins that progressively increased in abundance across development and was enriched for proteins associated with metabolic and catabolic processes, including organic substance metabolism, proteolysis, lipid-related pathways, and intestinal absorption. These changes are consistent with, but do not directly demonstrate, maturation of epithelial metabolic and absorptive functions during the transition from milk-dominant feeding to solid food exposure.

Cluster 2 displayed a biphasic pattern, with higher abundance early in life, a marked reduction around weaning, and partial recovery thereafter. The 128 proteins composing this cluster were enriched for small-molecule and organic acid metabolism, cellular respiration, fatty acid oxidation, and oxidative phosphorylation (Fig. 5c). These functions are associated with metabolic pathways in oxidative energy metabolism, prominent in the neonatal intestine, that are transiently downregulated during dietary and microbial transitions toward anaerobic metabolism and fermentation.

Cluster 3 included 492 proteins that were highly abundant shortly after birth and progressively decreased over time. This cluster was enriched for proteins associated with cytoskeletal organization, actin filament dynamics, nucleotide metabolism, and the tricarboxylic acid cycle (Fig. 5d). These enrichments are compatible with early postnatal tissue remodeling and high metabolic demand, but should be interpreted as associative signatures rather than direct evidence of specific developmental programs.

Cluster 4, composed of 95 proteins, exhibited a transient increase during early-to-mid development and was enriched for regulatory processes, including negative regulation of proteolysis and hydrolase activity, wound healing, blood coagulation, and responses to tissue injury (Fig. 5e), indicating dynamic regulation of tissue integrity during periods of rapid microbial expansion. To further characterize the physiological relevance of the temporal clusters, we examined host-derived proteins shared between cohorts for each cluster and identified conserved temporal patterns associated with immune signaling and gut barrier-related functions across developmental time points (Supplementary Figure S5).

Together, these findings show parallel developmental changes in host intestinal protein profiles and gut microbiome taxonomic and functional composition during early life.

### Expression of host antimicrobial peptides and carbohydrate-active enzymes during early life

To illustrate how global host proteome maturation translates into functionally relevant effector pathways at the host–microbiome interface, we focused on two biologically distinct classes of host proteins: antimicrobial peptides (AMPs), which have been implicated in host–microbiome interactions and microbial growth regulation [61, 62], and host-encoded carbohydrate-active enzymes (CAZymes), which modulate carbohydrate availability in the intestinal lumen [50].

**Fig. 6:**
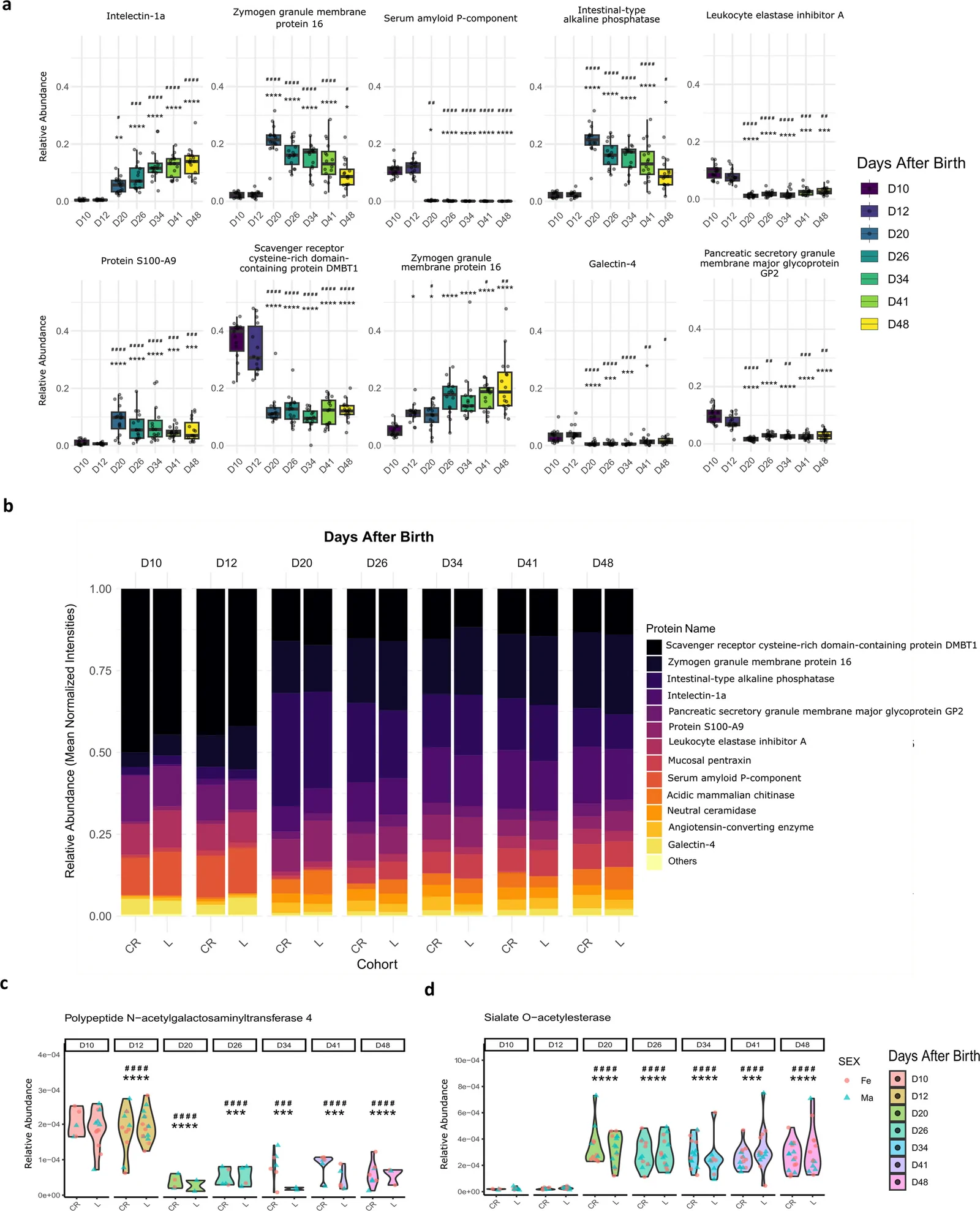
Temporal expression of antimicrobial peptides (AMPs) and carbohydrate-active enzymes (CAZymes) during postnatal early-life in local pups. **a** Relative abundance (presented as mean normalized relative abundances calculated as the summed peptide intensity for each protein divided by the total peptide intensity of the sample) of the top 10 most abundant AMPs. Estimated marginal means with Benjamin–Hochberg correction from Generalized Mixed-Effects Models including the fixed effects of Time, Cohort, SEX, and all their interactions (Time * Cohort * SEX), while accounting for repeated measurements within individuals (ID) and shared post-weaning housing conditions (cage_post) as random intercept effects detected a significant abundance difference over time (all significance levels are indicated in Supplementary Table 17). *n* = 16 mice (8 females and 8 males). **b** Relative AMP abundance between local and Charles River pups (local = L; Charles River = CR) over developmental time, as Others are aggregated into all proteins having less than 1% of the total summed intensity across the whole dataset. *n* = 16 mice for each cohort (8 females and 8 males). **c**–**d** Mean relative abundance of two carbohydrate-active enzymes: Polypeptide N-acetylgalactosaminyltransferase 4 and Sialate O-acetylesterase among the two cohort pups (local = L; Charles River = CR). Estimated marginal means with Benjamin–Hochberg correction from Linear Mixed-Effects Models including the fixed effects of Time, Cohort, SEX, and all their interactions (Time * Cohort * SEX), while accounting for repeated measurements within individuals (ID) and shared post-weaning housing conditions (cage_post), revealed significant temporal differences in CAZyme abundance. Statistically significant differences in AMPs and CAZyme abundance are indicated as follows: * compared to D10; # compared to D12. *p* < 0.05 (*, #), *p* < 0.01 (**, ##), *p* < 0.001 (***, ###), *p* < 0.0001 (****, ####). *n* = 16 mice for each cohort (8 females and 8 males)

Of the 152 annotated AMPs considered in our analysis [49], 90 were reproducibly detected in pups (Supplementary Table 17). A significant overall effect of Time on the abundance of the ten most abundant AMPs (representing 82% of the AMPs’ cumulative abundance) was detected in the mixed-effects model (F(6, 4590) = 2.20, *p* = 0.039), indicating that AMP abundance varies across developmental stages. (Supplementary Fig. 6a and Supplementary Table 16). Interestingly, these ten AMPs showed strong time-dependent dynamics (Fig. 6a).

No AMP was uniquely detected in either cohort, and no cohort- or sex-specific AMP repertoires were observed. Also, AMP composition and cumulative abundance were largely conserved between L and CR pups, with similar developmental trajectories across cohorts (Fig. 6b and Supplementary Fig. 6b).

Among the 5 host CAZyme categories described by Wardman et al. [50] (carbohydrate binding module, carbohydrate esterase, glycosyltransferase, glycoside hydrolases enzymes, and Polysaccharide lyase), 5 of 142 were detected, representing 0.14% of the host proteome detected in this study.

Host-encoded CAZymes displayed discrete and stage-specific expression patterns. Polypeptide N-acetylgalactosaminyltransferase 4 (Galnt4), an enzyme involved in glycoprotein glycosylation, was highly expressed during the neonatal period (D10–D12) and declined significantly thereafter in both cohorts (Fig. 6c). Glycoproteins constructed in the gastrointestinal tract heavily rely on GalNAc transferase family members, such as Galnt4, Galnt5, and B4glant2, which play crucial roles in modulating the intestinal epithelial development and maturation [63–65]. Conversely, host sialate O-acetylesterase showed a marked increase in expression from D20 onward (Fig. 6d). This enzyme, which regulates sialic acid availability and has been implicated in intestinal homeostasis and carbohydrate metabolism, increased in parallel with gut microbiome maturation and the expansion of microbial pathways associated with host-derived glycan utilization.

Together, these results delineate discrete developmental windows during which host antimicrobial and glycan-modifying activities are differentially deployed, highlighting parallel developmental changes in host effector functions and gut microbiome composition.

## Discussion

The metaproteomics workflow applied here [29], enabled reproducible detection of microbial taxa, host proteins, and low-abundance dietary proteins even at the earliest postnatal stages, when fecal sample amount and microbial biomass were highly limited. This analytical sensitivity was critical for resolving microbial, dietary, and host proteome axes from the same animals longitudinally assessed, and thereby capturing parallel developmental changes that would be difficult to assess with less sensitive approaches. Our study, based on repeated sampling of individual animals from postnatal day 10 onward, captured an integrative view of developmental transitions at a resolution previously missed in early-life studies [3–6, 66, 67]. Previous studies have described individual components of early-life gut maturation, including weaning-associated microbial succession, maternal-origin-associated effects, and functional microbiome profiling [15, 20, 68, 69]. However, many of these studies relied on 16S rRNA gene sequencing, metagenomics, or single-axis analyses, which primarily capture taxonomic composition or functional potential. The contribution of the present study is to integrate these processes longitudinally at the metaproteome level across microbial, dietary, and host axes. By resolving expressed microbial functions, dietary protein detection, and host intestinal proteome changes from the same samples, this study provides a coordinated reference framework for murine gut ecosystem development. This integrated design reduces confounding from cross-study comparisons and enables temporal alignment of dietary transition, microbial succession, functional redundancy, and host proteome maturation within a single experimental system.

### Early-life microbial community assembly dynamics and niche construction

Early-life gut communities were dominated by facultative anaerobes, particularly *Lactobacillus* species such as *L. murinus*, *L. reuteri*, and *L. johnsonii*. Metaproteomic profiles of these taxa were enriched for functions related to carbohydrate utilization and host–microbe interactions, consistent with hypotheses that early colonizers may contribute to modification of the neonatal gut environment [3, 70–75]. Such activities may be associated with changes in the physicochemical environment of the neonatal gut and with subsequent microbial succession [3, 9, 13]. These early colonizers were progressively replaced by obligate anaerobes, including *Bacteroides*, *Anaerotruncus*, and members of the Lachnospiraceae family. The similar structure of Bray–Curtis and Jaccard indices further supports the interpretation that developmental remodeling of the gut microbiota primarily reflects taxonomic succession. Thus, maturation was characterized not only by changes in the relative abundance of established taxa but also by the replacement of early-life species by later-stage adult-like taxa. Notably, this process was different between the two Cohorts for most of the experimental time points. This taxonomic succession coincided with weaning and is consistent with a transition toward a more anaerobic and metabolically complex gut environment occurring alongside dietary change and the increased representation of obligate anaerobes [3–6].

### Dietary transitions and early exposure to solid food

Direct detection of dietary proteins provided proteome-level evidence for time-dependent changes in dietary protein detectability during development. Valdés-Mas et al. estimated that dietary proteins represent less than 1% of the fecal proteome [49]. Milk-derived proteins (between 0.01% and 0.03% of the food proteins, as shown in Fig. 2b) were detected before weaning, whereas chow-associated proteins were detected at all the experimental timepoints. Given the very low abundance of dietary proteins, early detection of chow-associated proteins may reflect transient environmental or maternal-associated exposure (e.g., grooming or contact transfer) as well as limited incidental ingestion, rather than sustained dietary intake or ingestion of diet-derived material associated with coprography of maternal feces [12]. We additionally note that some plant-derived protein signals, particularly corn-associated proteins, may also partially originate from environmental exposure sources such as corncob bedding, which mice can occasionally interact with or ingest. Our sampling strategy via stimulation of fecal production through the anogenital reflex [39], in a sterile tube, reduces the possibility for cross-contamination with food, animal fur, and components present in the cage bedding. We note that food-derived proteins comprise only a very small fraction of the total fecal proteome; therefore, variation within this subset is more likely to reflect detectability and exposure dynamics than dietary contribution. Importantly, this temporal pattern was reproducibly detected across cohorts, sexes, and individuals by adopting a generalized linear mixed-effects modeling accounting for individual identity and cage effects, supporting a robust developmental trend in detectability.

### Functional redundancy and stabilization of the gut ecosystem

Although the observed increase in nFRp is consistent with progressive maturation and potential stabilization of the gut ecosystem, this interpretation should be made cautiously. The developmental increase in nFRp indicates a greater detectable overlap of expressed microbial functions across taxa during maturation. This pattern coincides with increasing taxonomic richness and functional complexity; however, it should be interpreted as an association rather than evidence that higher species richness directly drives functional redundancy. Because nFRp is derived from detected taxon–function relationships in metaproteomic data, it may also be influenced by several technical and biological factors, including higher microbial biomass in older animals, improved protein recovery from larger fecal samples, increased detectability of microbial proteins after weaning, and reference-database bias toward adult-associated taxa and functions. We therefore interpret increasing nFRp as being consistent with functional maturation of the gut ecosystem, rather than as definitive evidence of ecosystem stabilization. Thus, our data support a co-occurrence of increasing taxonomic diversity and the accumulation of overlapping functional capacity of the microbiome during development.

### Maternal origin and reproducibility of microbiome development

Comparison of pups born to long-established local colony mothers and pups born to newly purchased pregnant females revealed that maternal microbiota origin was associated with differences in engraftment trajectories [15, 16, 20, 76, 77]. Although maternal communities shared many species, each cohort harbored distinct taxa that may have influenced offspring microbiome assembly. Maternal species acquisition increased with age, and pups progressively converged toward their respective maternal profiles at both taxonomic and functional levels. Although L mothers harbored more cohort-specific species than CR mothers, L pups showed lower apparent maternal-source contribution over development. This argues against a simple explanation based on lower maternal richness or detectability in the L cohort. Instead, the data suggest that maternal-source contribution depends not only on the number of maternal taxa available but also on their ability to colonize, persist, or remain detectable in the offspring gut after weaning. Nevertheless, this analysis cannot fully separate engraftment efficiency from source-community composition, and we therefore interpret the metric as maternal-source contribution rather than direct engraftment efficiency.

Cohort-specific differences in engraftment persisted despite identical housing and dietary conditions, underscoring maternal microbiota as a key determinant of early-life microbial development[15, 16, 77]. At the same time, convergence of functional redundancy across cohorts suggests that distinct taxonomic trajectories can nonetheless yield similar ecosystem-level functional outcomes.

### Host intestinal proteome maturation and host–microbiome coupling

Host proteome analysis revealed conserved developmental trajectories that closely paralleled microbial community turnover. Early-life host protein profiles were enriched for pathways associated with epithelial growth and oxidative metabolism, consistent with rapid intestinal development in the neonatal period [66, 67]. In agreement with a previous study on mouse development adopting a metaproteomic approach [78], remarkable differences are detected from the early time point with maturation; host protein expression increased and shifted toward enhanced metabolic versatility, absorptive capacity, and regulation of tissue homeostasis. Notably, even subtle but significant shifts in host responses and microbiome composition–function profiles robustly separated the two cohorts over time, consistent with Pettersen et al. [69], highlighting tightly coupled host–microbe crosstalk during intestinal colonization. Because GO enrichment analyses are associative, we interpret these host-protein clusters as proteomic signatures consistent with developmental changes in intestinal metabolism, structure, and tissue regulation, rather than as direct evidence for activation of specific epithelial or immune developmental programs.

### Deployment of host effector pathways: AMPs and CAZymes

Host antimicrobial peptides showed stable overall abundance across development, sexes, and cohorts, but pronounced temporal reorganization of individual AMP profiles. Distinct subsets of AMPs predominated before versus after weaning, indicating compositional reprogramming rather than changes in total antimicrobial output.

Host-encoded carbohydrate-active enzymes displayed discrete, stage-specific expression patterns, with glycoprotein-modifying enzymes enriched early in life and enzymes regulating sialic acid availability increasing after weaning. These shifts reveal stage-specific changes in host effector protein expression at the host–microbiome interface. In parallel with the developmental changes observed in host CAZymes, microbial CAZyme profiles exhibited significant shifts over time in all functional categories except chitin/cell wall-associated CAZymes (Supplementary Fig. 6.c–i and Table 17), while Glycosaminoglycans Degradation became significantly more abundant at D48 in CR pups, suggesting a progressive maturation of microbial carbohydrate utilization capacity.

### Limitations and future directions

This study has several limitations. All mice used in our study belong to C57BL/6 strain. C57BL/6 mice are the most widely used model for microbiota-related human disease studies, given that they share approximately 11.8% of gut microbial species and 90% of functional pathways with humans [27, 79]. Importantly, the present study was performed exclusively in C57BL/6 mice under controlled laboratory conditions. Because gut microbiome assembly, immune development, diet, and developmental timing differ substantially between mice and humans, our findings should be interpreted primarily within the context of murine early-life development rather than as direct models of human microbiome maturation. Our experimental design comparing locally bred mothers to newly purchased animals acknowledges potential genetic or microbial drift. While our results show that overall developmental trajectories are preserved across cohorts, caution is warranted when translating specific taxonomic or functional differences to other mouse strains. The observed differences between cohorts should not be interpreted as direct proof of maternal microbiota transmission alone, but rather as cohort- or source-associated variance. Given the experimental design contrasting a long-established in-house colony with recently obtained pregnant females, these differences may reflect broader source-associated factors, including colony background, microbial drift, subtle genetic drift, prenatal environment, vendor-associated husbandry, transport stress, adaptation to the local facility, and post-weaning caging. Because metaproteomic detection sensitivity depends on sample, analytical, and database-related factors, the absence of protein detection does not necessarily indicate the absence of the protein or its biological function, especially for low-abundance features subject to increased quantitative uncertainty. Accordingly, the conclusions of the present study should be interpreted within the detectable metaproteome captured by the applied workflow. In addition, because corncob bedding was used throughout the study, the potential influence of compounds present in the bedding on mouse physiology and development should be considered when interpreting the results [80, 81]. Deeper fractionation, targeted enrichment strategies, or orthogonal validation approaches may further improve characterization of low-abundance proteins and functions. In general, taxonomic and functional annotation in metaproteomics remains constrained by reference databases [29, 82, 83], which limits a complete biological view of the gut development process. The confidence of taxonomy annotation remains a challenge in metaproteomics. Even though we applied a cut-off of a minimum of 3 species-specific peptides, the annotation is based on the available MetaLab database. Our study was designed as a longitudinal systems-level characterization of early-life gut maturation and should be interpreted primarily as an exploratory reference framework. Although microbial succession, functional redundancy, dietary protein transitions, and host intestinal proteome remodeling occurred along the experimental temporal windows, these parallel dynamics do not demonstrate causal relationships. Instead, they identify candidate periods and pathways in which host, dietary, and microbial processes are aligned during development. Mechanistic studies, including perturbation experiments, gnotobiotic transfer, cross-fostering, dietary manipulation, or targeted inhibition of candidate pathways, will be required to determine whether specific microbial taxa or functions actively drive host intestinal maturation. An additional limitation is that immune development was inferred only indirectly from fecal host proteins and pathway-level annotations. We did not perform direct immune phenotyping. Therefore, conclusions regarding immune maturation should remain cautious. Although both cohorts were raised under identical housing and dietary conditions after birth, and despite CR mothers being housed in our facility for several days before delivery, maternal-origin-associated effects may also reflect upstream factors occurring before cohort standardization, including vendor-associated husbandry conditions, prenatal exposures, transport and shipping stress, and adaptation to the local animal facility. Although both sexes followed shared maturation programs, we detected a limited but significant number of sex-specific changes. Our results open the door to more pronounced differences when considering menstrual cycle biology, therefore, supporting the recognition of sex as an important factor in mouse-based research [84]. Finally, while metaproteomics captures expressed functions, complementary measurements of microbial metabolites would further strengthen functional interpretation [85, 86].

## Conclusions

Our results provide an integrated view of gut microbiome and host intestinal co-variation during early life in mice. Our results expand our understanding of early-life host-microbiome interactions and highlight the importance of clearly modeling hierarchical structure and considering broader source effects when designing and interpreting experiments. These considerations are critical for improving robustness, reproducibility, and biological interpretation in mouse microbiome studies.

## Supplementary Information

Additional file 1: Supplementary Tables 1–18.

Additional file 2: Supplementary Figures 1–6.

## Data Availability

The datasets generated and/or analyzed during the current study are available in the PRIDE repository with the dataset identifier (PXD074520). Source data are provided with this paper.
