# Supplementary material for: Integrative characterization of host–microbiome-diet axes during early-life development of the murine gut
Source: Microbiome. 2026 Oct 6;14:250. doi: 10.1186/s40168-026-02541-3 (PMC13757233; doi:10.1186/s40168-026-02541-3)
Supplement: Supplementary file 1 [file 40168_2026_2541_MOESM1_ESM.zip › Supplementary Table 3.docx]

| Table 1. Mouse milk proteins list and corresponding Uniprot Entry code | |
| --- | --- |
| **Protein Name** | **Uniprot ID** |
| α_s1_-casein (α_s1_) | P19228 |
| β-casein (β) | P10598 |
| γ-casein (γ) | A0A0U1RP94 |
| kappa-casein | P06796 |
| Whey Acidic Protein (WAP) | P01173 |
| α-lactalbumin (α-lac) | P29752 |
| Apelin | Q9R0R4 |
| Lipid transferase | O70302 |
| Fatty acid desaturase 3 | Q9JJE7 |
| Alpha-S2-casein-like A | Q02862 |
| Lactoperoxidase | Q5SW46 |
| Lactoferrin | P08071 |
